# What Happens during the Stimulus Onset Asynchrony in the Dot-Probe Task? Exploring the Role of Eye Movements in the Assessment of Attentional Biases

**DOI:** 10.1371/journal.pone.0076335

**Published:** 2013-10-10

**Authors:** Kalina Petrova, Dirk Wentura, Christina Bermeitinger

**Affiliations:** 1 Department of Psychology, Saarland University, Saarbrücken, Germany; 2 Department of Psychology, University of Hildesheim, Hildesheim, Germany; University of Akron, United States of America

## Abstract

The dot-probe paradigm is one of the most often used paradigms to investigate attentional biases towards emotional information. However, a large number of the dot-probe studies so far used a long stimulus onset asynchrony allowing for eye movements to occur, which might increase the error variance. This study aimed at addressing this methodological issue by varying the instructions with regard to the gaze behavior and calculating the reaction time (RT) bias score (i.e., RTs for targets presented at the location of the emotional compared to the neutral stimulus) separately for trials with eye movements and trials without eye movements. Results of Experiment 1 (using typical instructions, i.e., instructions that are lenient with regard to eye movements) showed an RT bias, but only in the trials without eye movements The overall RT bias (calculated “blind” for eye movements) was non-significant. In Experiment 2, stricter instructions and small changes in the procedure led to a sharp decrease in the number of eye movements, such that both the RT bias in the trials without eye movements as well as the RT bias across all trials was significant.

## Introduction

A frequently used paradigm to investigate selective attention towards emotional information, particularly in experimental psychopathology, is the dot-probe paradigm (see [1], for a review). In this paradigm, participants are typically presented with two stimuli, one neutral and one emotional, simultaneously side by side for a brief period of time. Subsequently, a probe appears either in place of the neutral stimulus (*invalidly cued* condition) or in place of the emotional stimulus (*validly cued* condition), and participants have to categorize it according to a spatially unrelated dimension (e.g. [2]). The rationale in this paradigm is similar to the one in the exogenous spatial cueing paradigm, namely that participants should be faster in trials in which the probe appears in the validly cued (viz. attended) location than in trials in which the probe appears in the invalidly cued (viz. unattended) location (see [3]). Thus, faster reaction times (RTs) in validly cued than in invalidly cued dot-probe trials (i.e., an RT bias) are interpreted as emotion-related attentional bias.

However, there is one important caveat. Compared with cueing studies in basic cognitive research, a rather long stimulus onset asynchrony (SOA) of cues and probes (i.e., 400–1250 ms) has typically been used in dot-probe studies. Therefore, the RT bias could be attributed to either enhanced vigilance or delayed disengagement of attention (e.g. [4], [5]). Furthermore, basic research with the cueing paradigm indicates that such long SOAs are associated with longer RTs for validly cued trials compared with invalidly cued or neutral trials (‘inhibition-of-return’ effect; [6]). Moreover, such long SOAs are sufficient for the programming and execution of at least one eye movement. Thus, the dot-probe paradigm can measure *covert* shifts of attention (i.e., changes in the focus of attention that took place without eye movements) as well as *overt* orienting of attention (i.e., eye movements). Whereas, however, in basic cueing studies either very brief SOAs (precluding eye movements) are used, or only trials in which participants made no eye movements enter into the analyses in order to make sure that the effects found are not due to overt orienting, in dot-probe studies no differentiation is typically made between covert and overt attention.

From the perspective of someone interested in attentional biases towards emotional stimuli one might ask why it is important to control for the occurrence of eye movements in the dot-probe task given that gaze shifts closely follow shifts in covert orienting (e.g. [7]). First, previous studies have shown prolonged reaction times when the responses were given shortly after a saccade compared to the condition in which no saccade preceded the response (e.g. [8]; see also [9]). This finding has been attributed to an inhibitory interaction between the saccades and the manual responses in the afferent system or at the motor control site [8]. Thus, one might argue that in dot-probe trials in which an eye movement occurred just before the probe onset, the reaction time effects diminish and the dot-probe effects are solely based on the trials in which no eye movements occurred. Alternatively, given the strong relationship between covert and overt attention, one might argue that in trials in which an eye movement was directed towards the emotional stimulus a positive RT bias score results (i.e., faster reaction times in validly cued trials compared to invalidly cued trials), whereas in trials in which an eye movement was directed towards the neutral stimulus a negative RT bias score results (i.e., faster reaction times in invalidly cued trials compared to validly cued trials). In any given case, it is important to investigate which trials underlie the RT bias effect (i.e., those in which no eye movements occurred or those in which an eye movement was directed towards the emotional stimulus) or whether both types of trials lead to similar effects.

Second, long SOAs (i.e., >300 ms) are usually associated with the phenomenon of inhibition of return (see above). Undoubtedly, there is strong evidence suggesting that covert attention is biased towards emotional information with SOAs below 100 ms, as the rare studies using such short SOAs indicate (e.g. [2], [10]). There is also some evidence suggesting that covert orienting is biased towards emotional information even with long SOAs. For example, [9] found that some participants made a lot of eye movements whereas others only rarely directed their gaze towards the cues. Those who made few eye movements had faster RTs than those who made a lot of eye movements. Whether or not participants made a lot of eye movements did not modulate the effect of trait anxiety on the the RT bias score (i.e., larger RT bias score in the high and medium anxious than in the low anxious participants). Thus, the authors concluded that the RT bias was independent of the extent of overt orienting. However, no study has yet separated the effects of overt and covert orienting on the RT bias in the dot-probe task at the level of individual trials.

Third, currently there is evidence that eye movements in the dot-probe paradigm are directed with higher probability towards threat-related information [9], [11], [12]. Assume, for example, that in 55% of the trials with eye movements the eye movement was directed towards the emotional stimulus (i.e., in 45% of the trials with eye movements the eye movement was directed towards the neutral stimulus). This value (if significantly deviant from 50%) would support the general bias hypothesis. However, it is unclear whether this effect directly translates into an RT bias. Assume, for example, that on average an eye movement towards a stimulus is associated with a facilitation of 20 ms in RTs if the dots replace that stimulus compared to the dots replacing the other stimulus. Then, the bias towards threat might be statistically non-detectable in the RTs because only 55% of the trials are associated with an average bias of +20 ms while 45% of them are associated with the reversed difference. Thus, the effects of the two sub-samples of trials (i.e., the trials with an eye movement towards the neutral stimulus and the trials with an eye movement towards the emotional stimulus) might almost cancel out each other (0.55 × 20 ms +0.45 × −20 ms = 2 ms).

As already indicated above, an often used method to take the occurrence of eye movements into account is the use of short SOAs that do not allow eye movements to take place (e.g. [2], [10], [13]). As an alternative, [14] presented simultaneously with the stimulus pair a number in the center of the screen which participants had to read aloud. Other studies presented the fixation cross throughout the whole trial and instructed participants to maintain their gaze on it [15]–[17]. However, although in recent years some researchers have considered the problem of long SOAs and possible influences of eye movements, the bulk of the dot-probe studies used long SOAs and ignored this issue. In their review, [1] coded dot probe studies for “exposure time” of stimuli (subliminal, 500 ms, ≤1000 ms). “Exposure time” is of course not identical with SOA, but can plausibly be seen as the lower limit of SOA in this paradigm. Thus, even for some studies coded as “subliminal” with regard to exposure time, the SOA might be quite long. Of all dot probe studies that were coded for this feature (n = 101), 85% were coded as either “500 ms” or “≤1000 ms”. Since [1], we found in total 251 experiments (published in 198 articles); still 67% used an SOA of 400 ms or more.

The aim of our studies was to retrospectively relate to the former studies by examining the role of eye movements in long SOA versions of the dot-probe paradigm. The importance of this endeavor is given by the fact that the evidence provided by published dot-probe studies in general – including older ones – leads to prevailing conclusions that attentional bias towards threat-related information is reliably demonstrated only in high anxious participants, but not in unselected samples (e.g. [1]). As argued above, however, it might be possible that previous studies found no evidence for an attentional bias with unselected samples because they did not control for the occurrence of eye movements.

In this study, we computed RT bias scores for each participant (1) across all trials (overall RT bias), (2) across the trials in which participants made no eye movements during the presentation of the stimulus pair (covert RT bias), and (3) across the trials in which participants made eye movements during the stimulus pair presentation (overt RT bias). This allows for an analysis of whether RT bias scores in the dot-probe paradigm are influenced by the occurrence of eye movements.

Experiment 1 investigated the effects of the type of orienting (overt vs. covert) on the RT bias under conditions that are typical for dot-probe studies (i.e., no specific gaze behavior instructions were given to the participants), and thus should allow for a relatively high number of eye movements. In Experiment 2, the effects of overt and covert attention on the bias were examined under experimental conditions that aimed to reduce the number of eye movements and thus, make the experiment in this regard more similar to a basic cueing experiment. Because the focus of these experiments was on methodological issues, we used pairs of angry and neutral faces only and did not address the topic of emotional discriminability (i.e., whether any negative or even any emotional stimulus triggers attentional shifts).

## Experiment 1

### Method

#### Ethics statement

All participants provided their written informed consent to participate in the study. The study (including the consent procedure) was approved by the Ethics Committee of the Faculty 5 Empirical Social Sciences of Saarland University.

#### Participants

The sample in Experiment 1 consisted of 21 (16 female) non-psychology students from Saarland University, Germany. Median age was 23 years (range from 21 to 28 years). All had normal or corrected-to-normal vision. They were paid for their participation.

#### Apparatus and material

The neutral and angry face photographs of 20 individuals (10 female) from the Karolinska Directed Emotional Faces Set were used [18]. This resulted in 40 face photographs. The face pairs that were presented as cues consisted always of one neutral and one angry face. The photographs did not differ in the mean values of their luminance histograms as created by Adobe Photoshop, *t*(38) = 1.12, *p* = .27. The experiment was run using the E-Prime software and a 17′′ CRT monitor. Eye movements were recorded using an SMI iView X Hi-Speed eye-tracker with a temporal resolution of 500 Hz and a spatial resolution of 0.01°.

#### Design

Essentially, we varied whether the probe appeared at the location of the angry face (validly cued condition) or the neutral face (invalidly cued condition). In detail, we manipulated three experimental variables within-participants: the location of the angry face (left vs. right), the location of the probe (left vs. right), and the probe type (: vs..). As main dependent variable we used the RT bias score (i.e., attentional bias; dot probe effect) which is computed as difference in RTs of invalidly cued trials and validly cued trials.

#### Procedure

Participants first completed the informed consent form. Viewing distance was 64 cm. Head movements were restricted by a forehead and chin rest. The individual eye-tracker adjustments were performed followed by a 9-point-calibration. Subsequently, the instructions were given on the screen. There were 160 experimental trials, such that each face appeared once in each condition (probe location × probe type). The faces were randomly assigned with the constraint that the angry picture of a given person was never paired with the corresponding neutral picture. There were 10 practice and 10 buffer trials before the experimental trials. Each trial started with a drift-correction point that appeared at the center of the screen followed by a fixation cross that was presented for 1000 ms. Participants were instructed to fixate both of them. Subsequently, the face pair was presented for 400 ms. Participants were told that the pictures were irrelevant for their task. The photographs were presented side by side. The size of each photograph was 5.2 cm×7.2 cm (distance of inner edges was 4.8 cm). Directly after the face pair, the probe appeared either at the location of the angry face (validly cued trial) or at the location of the neutral face (invalidly cued trial), and remained there until participants responded. Participants were instructed to quickly and accurately categorize the probe by pressing the ‚F‘ key if it consisted of two horizontal dots or the ‚J‘ key if it consisted of two vertical dots.

### Results

#### Data preparation

Data from trials with errors (3.04%) and outlier RTs (3.15%) were discarded. Outliers were RTs faster than 200 ms, slower than 1500 ms, or RTs that were 1.5 interquartile ranges above the third quartile of the individual distribution [19]. Eye movement data were prepared in BeGaze (SensoMotoric Instruments, Teltow/Berlin, Germany), where saccades were defined as each eye movement whose peak velocity was greater than 30°/sec. An alpha-level of 5% was adopted for all tests; *p*-values refer to two-tailed testing, unless otherwise noted.

#### Overall RT bias

**Figure 1:**
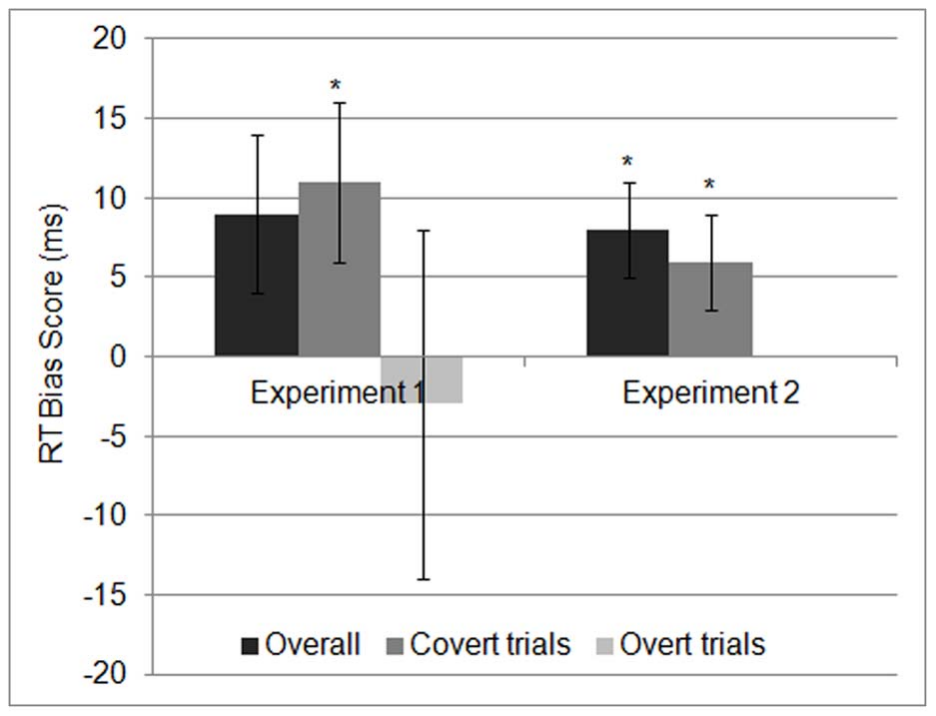
Difference in mean RTs between validly cued and invalidly cued conditions (in ms) across all trials (overall), across trials in which participants made no eye movements during the presentation of the face pair (covert trials), and across trials in which participants made any eye movement towards one of the faces during their presentation (overt trials) in Experiment 1 and Experiment 2 (error bars represent the standard error of the mean; * *p*<.05). Positive values indicate an attentional bias towards threat (i.e., faster RTs in validly cued compared with invalidly cued trials).

The RT bias was computed for each participant by subtracting the mean RT in the validly cued conditions from the mean RT in the invalidly cued conditions (see Table 1 for mean RTs). Aggregation for validly cued and invalidly cued conditions was unweighted regarding the balancing scheme. That is, we first calculated mean RTs for the 2 (cueing) × 2 (probe location) × 2 (probe type) cells to collapse then over probe location and probe type for validly cued and invalidly cued conditions. We ran the complete 2×2×2 ANOVA and found no significant main effects or interactions with the probe type or probe location factor, except of the interaction of probe location and probe type, *F*(1,20) = 95.05, *p*<.001, indicating faster RTs if response type [left key vs. right key] is compatible with the probe location compared with non-compatible conditions (i.e., Simon effect), all other Fs <1, except the main effect of probe type, *F*(1,20) = 3.41, *p* = .08. Thus, the RT bias score is not confounded with the Simon effect (see [20]; i.e., faster RTs if response type [left key vs. right key] is compatible with the probe location compared with non-compatible conditions). Numerically, the mean RT bias was positive (*M* = 9 ms, *SD* = 22.9; see Figure 1). However, it was not significantly above zero, *t*(20) = 1.75, *p* = .10, *dz* = .38. Note, that there was one outlying value for the RT bias. Excluding this participant yielded an RT bias of *M* = 5 ms (*SD* = 15 ms), *t*(19) = 1.48, *p* = .16, *dz* = .33.

**Table 1:** Mean RTs for validly cued and invalidly cued conditions (in ms; *SD* in parenthesis) across all trials (overall), across trials in which participants made no eye movements during the face pair presentation (covert) and across trials in which participants made any eye movement towards one of the faces (overt).

	Valid cuing	Invalid cuing
*Experiment 1*		
Overall	471 (57.6)	480 (71.3)
Covert	467 (57.4)	478 (68.1)
Overt^a^	491 (66.8)	488 (79.3)
*Experiment 2* b		
Overall	533 (81.6)	541 (86.6)
Covert	532 (80.9)	538 (84.9)

a RTs in the overt trials in Experiment 1 are adjusted for the Simon effect.

b Due to the low number of overt trials in Experiment 2 mean RTs for overt trials were available for only 11 out of 19 participants.

#### RT biases depending on the type of orienting (covert vs. overt)

To examine the RT bias score as a function of the type of orienting we divided the trials into two sets depending on whether participants’ gaze remained fixed during the face pair presentation or whether participants made any horizontal eye movements during that time. In the majority of the trials (*M* = 75.27%) participants made no eye movements during the face presentation while fixating the centre of the screen (*covert trials*, 32.50%–92.50%, *SD* = 17.04%, across participants). As *overt trials*, we took trials in which the first eye movement had a latency slower than 80 ms and shifted in horizontal direction towards one of both faces (*M* = 11.43%, 0.63%–33.75%, *SD* = 10.39%, across participants). Thus, we discarded (1) trials with anticipatory eye movements (i.e., eye movements with latencies faster than 80 ms, *M* = 2.65%, 0%–11.25%, *SD* = 3.42%, across participants), (2) trials in which the first eye movement shifted in vertical direction (*M* = 2.11%, 0%–11.88%, *SD* = 3.05%, across participants), and (3) trials in which the fixation preceding the first saccade was not on the center of the screen (*M* = 2.35%, 0%–7.50%, *SD* = 2.06%, across participants). RTs in the covert trials (*M* = 473 ms) were significantly faster than RTs in the overt trials (*M* = 488 ms), *t*(20) = 2.80, *p* = .01¸ *dz* = .61.

We calculated the covert RT bias score by subtracting the mean RT in the validly cued conditions from the mean RT in the invalidly cued conditions across the trials without eye movements during the face pair presentation period (see Table 1 for mean RTs). Now, the RT bias score (*M* = 11 ms, *SD* = 20.7) was significant, *t*(20) = 2.39, *p* = .03, *dz* = .52, showing significantly faster RTs in the validly cued than in the invalidly cued conditions (see Figure 1). (Excluding again the participant with the outlying value – see above – yielded *M* = 8 ms (*SD* = 15 ms), *t*(19) = 2.26, *p* = .04, *dz* = .51.).

Similarly, we calculated the overt RT bias score by subtracting the mean RT in the validly cued conditions from the mean RT in the invalidly cued conditions across the trials with horizontal eye movements during the face pair presentation. Due to the low number of trials, we were faced with missing data in the 2 (cueing) × 2 (probe location) × 2 (probe type) data matrix of some participants. Therefore, we adjusted for the Simon effect (see above) in the following way. We first calculated the individual Simon effect for each participant across the covert trials (i.e., mean RT in incompatible covert conditions minus mean RT in compatible covert conditions divided by two). This constant was added to the compatible conditions and subtracted from the incompatible conditions (if they were not missing) before collapsing RT means of the validly cued and invalidly cued conditions. The overt RT bias score did not significantly differ from zero, *M* = −3 ms (*SD* = 45 ms), *t*(16) = 0.27, *p* = .79, *dz* = .07. To examine whether the RT bias score depended on the eye movement direction, the overt trials were divided into trials in which the first saccade was directed towards the angry face (*M* = 6.28%, 0.63%–17.50%, *SD* = 5.56%) and trials in which the first saccade was directed towards the neutral face (*M = *5.15%, 0%–16.25%, *SD* = 4.96%). Although both RT bias scores were not significantly different from zero – *M* = 20 ms (*SD* = 57 ms), *t*(13) = 1.30, *p* = .22, *dz* = .35, for trials with the first saccade directed towards the angry face, *M* = −21 ms (*SD* = 66 ms), *t*(12) = 1.16, *p* = .27, *dz* = .32, for trials with the first saccade directed towards the neutral face –, numerically they were face-valid: If the location of the probe was identical to the location targeted by the saccade, RTs were faster.

#### Eye movements

To see whether eye movements were influenced by the face type, an eye movement bias score (EM bias score) was calculated [9], [11]. The EM bias score was calculated by dividing the number of trials in which the first saccade was directed towards the angry face by the total number of trials in which the first saccade was directed towards one of the two faces. Thus, a score greater than.5 suggests a tendency to look first at the angry face rather than the neutral one. The mean EM bias score found was *M* = .61 (*SD* = .19), which was significantly larger than.5, *t*(20) = 2.70, *p* = .01, *dz* = .59.

### Discussion

Participants in this experiment made eye movements in 11.43% of the trials. Roughly, this result is in accordance to what was found by [9]. Our study, however, goes beyond the findings from [9] since we analyzed RT bias effects separately for trials with eye movements and trials without eye movements. Whereas the covert RT bias score was significantly positive (i.e., in covert trials, participants were faster in responding to probes that replaced the angry face than to probes that replaced the neutral face), the overt one did not differ from zero. Note that in concordance to the covert RT bias score the eye movements themselves were biased towards the angry face – participants initially moved their gaze with higher probability to the angry face compared to the neutral one – and that the RT bias score was numerically positive as well if calculated across the trials in which the eye movement was directed towards the angry face (20 ms). The sub-sample of overt trials in which the eye movement was directed towards the neutral face seemed to decrease the overall RT bias score to a non-significant level. Not surprisingly, in those trials the RT bias score was numerically negative (−21 ms) – RTs were faster if the location of the dots was identical to the location targeted by the first saccade. Most importantly, however, since the ratio ‘number of eye movements towards angry’-to-‘number of eye movements towards neutral’ was only (approximately) 60∶40, the negative RT bias score across the trials in which the first saccade was directed towards the neutral face almost cancelled out the positive RT bias score found across the trials in which the first eye movement was directed towards the angry face. This resulted in a non-significant overt RT bias score, which on its part caused a non-significant overall RT bias score. Therefore, ignoring the problem related to the eye movement occurrence reduces the power of dot-probe experiments.

## Experiment 2

The aim of Experiment 2 was to reduce the number of eye movements made during the face pair presentation by (1) giving stricter fixation instructions to the participants and (2) leaving the fixation cross visible throughout the cue presentation. Of course, other techniques allow stricter avoidance of eye fixations on the cues (e.g., subliminal presentation duration or gaze-contingent masking procedure; [21]). Our study however aimed at referring to the typical dot-probe studies, many of which still use long SOAs and presentation duration as mentioned above. Therefore, we kept the SOA correspondingly long. We did not use a gaze-contingent masking technique since this would have thoroughly changed the paradigm.

### Method

#### Participants

The sample in Experiment 2 consisted of 19 (15 female) non-psychology students from Saarland University, Germany. Median age was 24 years (range from 20 to 35 years). All had normal or corrected-to-normal vision. They were paid for their participation.

#### Apparatus, Material, Design, and Procedure

Everything was the same as in Experiment 1 except for the following changes. To reduce the number of eye movements a gaze-contingent control at the beginning of each trial was introduced. The trial started only after the participant had fixated the fixation cross for 1000 ms. As soon as the subject did not fixate the fixation cross, a reminding message was shown and the fixation cross was presented again. If this message appeared three consecutive times, a drift correction was automatically performed. Thereafter, the fixation procedure started again. In addition, the fixation cross was presented continuously from trial onset until face pair offset. Here, the *gap effect* should be pointed out [22]. In the context of the antisaccade paradigm, in which participants are instructed to respond to a peripheral stimulus with a saccade in the opposite direction [23], the gap effect refers to the finding that the disappearance of the fixation cross shortly before the peripheral stimulus appears increases the probability of executing a saccade towards the stimulus and decreases the saccade latencies [24]. In the context of the dot-probe paradigm, the relatively high number of eye movements can be attributed to the fact that the fixation cross disappears before cue onset. Thus, the number of eye movements is likely to decrease if the fixation cross remains visible during the cue presentation.

### Results

#### Data preparation

The eye movement data were prepared in the same way as in Experiment 1. For the RT data, trials with errors (4.21%) and outlier RTs (4.51%) were discarded.

#### Overall RT bias

As in Experiment 1, the RT bias score was first computed across all trials by subtracting the mean RT in the validly cued conditions from the mean RT in the invalidly cued conditions (see Table 1 for mean RTs). We ran the complete 2×2×2 ANOVA and found no significant main effects or interactions with the probe type or probe location factor, except of the interaction of probe location and probe type, *F*(1,18) = 42.49, *p*<.001, indicating faster RTs if response type [left key vs. right key] is compatible with the probe location compared with non-compatible conditions (i.e., Simon effect), all other Fs <2.02, except the main effect of probe type, *F*(1,18) = 3.67, *p* = .07. Similarly to Experiment 1, the RT bias score was numerically positive (see Figure 1). This time, however, it was clearly significant, *M* = 8 ms, *SD* = 12.3, *t*(18) = 2.91, *p* = .01., *dz* = .67.

#### RT biases depending on the type of orienting (covert vs. overt)

In 85.95% of the trials participants’ gaze remained fixed on the fixation cross during the face pair presentation (*covert trials*, 61.88%–93.75%, *SD* = 6.98%, across participants). The mean number of *overt trials* (i.e., trials in which the first eye movement had a latency slower than 80 ms and shifted in horizontal direction towards one of both faces) was 2.93% (0%–16.88%, *SD* = 4.01%, across participants). Data from (1) trials with anticipatory eye movements (*M = *1.18%, 0%–3.75%, *SD* = 0.97%, across participants) and (2) trials in which the first eye movement had a latency greater than 80 ms but shifted up or down (*M* = 1.22%, 0%–5.00%, *SD* = 1.37%, across participants) were discarded. The RTs in the covert trials were significantly faster (541 ms) than the RTs in the overt trials (639 ms), *t*(15) = 5.24, *p*<.001, *dz* = 1.31.

The covert RT bias score was computed by subtracting the mean RT in the validly cued conditions from the mean RT in the invalidly cued conditions across the covert trials (see Table 1). As Figure 1 shows, it was significantly positive, *M* = 6 ms, *SD* = 11.3, *t*(18) = 2.43, *p* = .03, *dz* = .56.

Due to the low number of trials, the RT bias score for overt trials could be calculated only for roughly half of the sample. In addition, even for these participants the high number of missing values impeded a valid analysis. Therefore, we refrained from further analysing this score.

#### Eye movements

Again, a more differentiated eye movement analysis was made. These findings, however, should be considered with caution given the small number of overt trials (2.93%). The EM bias score was computed in the same way as in Experiment 1. This resulted in a mean EM bias score of.62 (*SD* = .33), which failed to be significantly different from.5, *t*(15) = 1.48, *p* = .08 (one-tailed), *dz* = .37, probably due to power restrictions.

#### Comparison across experiments

Experiment 1 and 2 differed with regard to the mean RTs (see Table 1). This difference was significant, *t*(38) = 2.63, *p* = .01, *dz* = .83. The corresponding analysis for errors was not significant, *t*(38) = 1.32, *p* = .19, *dz* = .42. The RT biases (overall and covert) as well as the EM bias were not significantly different between the experiments, all *t*s <1. (This even holds after exclusion of the outlying value in the RT bias in Experiment 1; see above.).

### Discussion

The manipulations taken to reduce the number of eye movements in Experiment 2 were successful in that they caused a considerable decrease in the number of overt trials (from approximately 11% in Experiment 1 to approximately 3% in Experiment 2). As a consequence, the (low number of) overt trials in which the first eye movement was directed towards the neutral face did not considerably reduce the power of the test for the overall RT bias score (i.e., the bias score calculated across all trials, irrespective of eye movements), which was significant as well.

Of course, reduction of the number of trials in which overt shifts occurred does not allow for the same fine-grained analyses as in Experiment 1 due to a power loss and sharply increased number of missing observations. Given this backdrop, it is interesting to note that the EM bias score (i.e., the bias towards first moving the gaze to the angry face) was numerically comparable to the one in Experiment 1. Though it was not significantly different from.5 (with *p* = .08, one-tailed), the result seems to be a corroboration rather than a falsification of the claim that eye movements are biased.

## General Discussion

In two dot-probe experiments the effect of the eye movement occurrence on the RT biases was investigated. These experiments were the first which investigated the eye movements during a dot-probe task in a fine-grained trial-based manner.

The first finding was that participants overall showed a lower number of eye movements when they were strictly instructed to restrain their eye movements (Experiment 2) compared with when they did not receive any specific gaze behavior instructions (Experiment 1). Second and more important, the RT bias was only significant if the trials in which eye movements occurred were excluded (Experiment 1), or if participants showed only a low number of trials with eye movements (Experiment 2). Third, participants’ initial gaze was directed with higher probability to the angry face than to the neutral one.

Thus, our experiments result in three conclusions. First, a valid attentional bias effect (i.e., the RT bias) in the dot-probe paradigm might remain undetected if procedural details (i.e., gaze behavior instructions and continuous fixation cross presentation) do not lead participants to a strict fixation behavior. In Experiment 1, the RT bias score was non-significant if calculated ‘blind’ regarding the eye movement occurrence due to the number of trials in which the first eye movement was directed towards the neutral face. In those trials participants were faster if the probe appeared on the location of the neutral face than if it replaced the angry face (i.e., a negative RT bias score resulted). In contrast, the RT bias score was significantly positive if we restricted the analysis to trials without eye movements (i.e., the valid trials regarding the underlying rationale of covert attentional movements).

Admittedly, in terms of milliseconds, the RT biases are quite small. Moreover the advantage of Experiment 2 seems to be quite restricted. But note that the effect sizes more clearly show what is going on: The overall RT bias was about twice as large in Experiment 2 compared to Experiment 1, whereas the covert biases were roughly comparable. In this regard it is interesting to note that [1] reported an average bias of *d* = .37 for anxious participants (see also below). In our experiments, the effect sizes were between *d = *.51 and.61 for the covert biases.

The results of this study suggest that the validity of the dot-probe paradigm might be enhanced by taking into account methodological issues that influence the number of eye movements made during the presentation of the stimulus pair. [1] suggested that attentional bias towards threat-related information is reliably demonstrated only in high anxious participants. We have to admit that we did not screen our samples for cases of high trait anxiety. That is, our finding of a significant overall RT bias with a non-selected sample (in Experiment 2) is, strictly speaking, not in contradiction to the conclusion made by [1]. However, it seems unlikely that our sample included a high number of participants with high trait anxiety that caused the overall effect. We interpret this as evidence for the fact that attentional biases indeed exist in non-selected samples as well (i.e, in samples with low anxiety on average), given some constraints as realized in Experiment 2. Nevertheless, it would be interesting to investigate RTs and gaze behavior of high anxious vs. low anxious participants during dot-probe tasks using different instructions and procedures regarding gaze behavior. In particular, it would be interesting to investigate whether the instructions and procedural details used in Experiment 2 exert a general increasing effect on attentional biases.

Second, simple variations regarding instructions and procedural details sharply reduced the number of eye movements. Thus, the RT bias score now was significant even if calculated ‘blind’ regarding the occurrence of eye movement (Experiment 2).

Third, eye movements were biased towards the angry face. This result occurred in both experiments (though it was, albeit not surprisingly, non-significant in Experiment 2 due to power reasons) and is, thus, consistent with the results by [9] and [11]. Importantly, these results suggest that covert attention was biased to the emotional stimulus followed by a corresponding eye movement. Thus, it is plausible that both effects, that is, the RT bias in the covert trials (i.e., the trials without eye movements) as well as the eye movement bias reflect the same basic process. That is, we might be faced by a tradeoff in dot-probe experiments: If we allow for a considerable number of eye movements, a considerable number of eye movements towards the neutral face occur as well. Thus, the covert bias effect might be weakened because in those trials RTs are faster when the probe appears in place of the neutral face (invalidly cued trials) than the place of the angry face (validly cued trials); if we do not allow for eye movements, the EM bias as reflected by the probability of first saccading towards the angry face is weakened. In any given experiment the balance might be very unfavorable such that both biases are nonsignificant.

Finally, it should be noted that the RTs in Experiment 1 were considerably faster than the RTs in Experiment 2. One possible reason for this difference might be the stricter fixation instructions in Experiment 2 as well as the procedural changes (i.e., the continuous presentation of the fixation cross and the gaze-contingent control technique in the beginning of each trial in Experiment 2). In particular, similarly to saccade latencies, RTs have been found to accelerate when a temporal gap was inserted between the fixation cross presentation and the target display compared to the condition in which the fixation cross was continuously presented (e.g. [25], [26]). The gap effect has been attributed to warning mechanisms in terms of unspecific activation of all sensory and motor processes that are required for a certain task (e.g. [27]) and attentional disengagement (e.g. [28]).

In conclusion, we can note that by using a rather long SOA (i.e., 400 ms) – which is typical for a lot of dot probe studies (see above) but which is vulnerable for the occurrence of eye movements and does not allow an interpretion in terms of fast acting attentional mechanisms – we found RT biases towards emotional material in samples which are not selected for anxiety. Either we discarded trials in which participants made eye movements (Experiment 1 without strict instructions regarding eye movements) or we sharpened the instructions asking the participants to avoid eye movements, introduced gaze-contingent control of eye-movements and presented the fixation cross during the whole trial (Experiment 2). Thus, it seems that given some constraints attentional biases in non-selected samples – whose existence was largely questioned – can be possibly discovered. It might be that eye movements cover the attentional biases in non-selected samples. Additionally, the longer the SOA, the more probable are eye movements. In turn, it seems justified to recommend stricter control of eye movements (either by instructions, procedural details, or by use of eye tracking) if one wants to explore attentional biases in non-selected samples.
